# Effects of Particle Shape and Surface Structure on the Adsorption Properties of Polystyrene Microplastics

**DOI:** 10.3390/polym16223159

**Published:** 2024-11-13

**Authors:** Natalia Shevchenko, Olga Iakobson, Vladimir Isakov, Ivan Zorin

**Affiliations:** 1 Microplastics Research Center, Yaroslav-the-Wise Novgorod State University, B. St. Petersburgskaya Str. 41, 173003 Veliky Novgorod, Russia; 2 Department of Chemistry, Saint Petersburg State University, University Av., 26, 198504 Saint Petersburg, Russia

**Keywords:** polystyrene microspheres, spherical and non-spherical particles, adsorption, surface layer structure, porosity

## Abstract

Model spherical polystyrene particles are studied to understand the interactions of microplastics with organic pollutants. Analysis of the experimental results presented in the literature is complicated since researchers use different types and concentrations of particles, durations of tests, etc. In addition, there is little information on the effect of the structure of the surface layer of polystyrene particles on the processes under study, and the question of the effect of the shape of polystyrene particles remains open. Here, we present the first results of a model experiment to study the effect of the shape and structure of the surface layer of polystyrene microspheres and non-spherical particles of 2 to 5 μm in size on the sorption properties in relation to model molecules of rhodamine B as a model organic pollutant. The properties of both the initial model polystyrene particles and the modified ones were studied by optical, transmission electron, and atomic force microscopy, as well as using the Brunauer–Emmett–Teller method (BET). The sorption process was studied by spectrophotometry, and the analysis of sorption curves was carried out using the Langmuir model. It is shown that the shape of polystyrene model particles does not have a significant effect on the sorption capacity. At the same time, the sorption processes of rhodamine B molecules are determined by the structure of the surface layer, which can be changed, for example, by exposing the polystyrene microspheres to N,N′-dimethylformamide.

## 1. Introduction

The problem of microplastics and their impact on the environment is currently a topic of much research [1,2,3,4,5]. This is due to the fact that microplastic particles are found in the water, soil, and living organisms [6,7,8]. At the same time, the search for microplastics in the environment is a complex and not always unambiguously solvable problem [9,10,11], since microplastic particles found in nature are characterized by different shapes and sizes, as well as origins (synthetic or natural). Today, researchers are making efforts to study the impact of microplastics on the environment using two approaches. The first approach includes searching for and establishing the patterns of the impact of “real” microplastics on environmental matrices and living objects. However, microplastics in the environment are complex polymer systems that must first be isolated and characterized. In the second approach, model microplastic particles of various natures and sizes are synthesized and experimental conditions for the impact of such particles on cells and species (viability of blood cells, vital activity of fish, etc.) are simulated [12,13,14,15,16,17,18].

Conducting large-scale studies on the impact of synthesized model microplastic particles will help researchers to understand and predict the impact of “real” microplastics on processes occurring in wildlife. Most studies published to date indicate that polystyrene particles were often used as model polymer particles. However, the analysis of experimental results is complicated, since different experimental parameters have been adopted (particle dimensions and concentrations, duration of biological tests, environmental conditions, etc.). Moreover, the interaction of microplastic with various environmental compounds does not only depend on the particle size, and a thoughtful analysis requires considering the effects of the microplastic’s surface structure. In fact, methods for the preparation of spherical polystyrene particles of different sizes ranging from 30 nm to hundreds of microns are well-known, but even minor changes in the synthesis conditions lead to the formation of polymer particles of comparable diameters, but differing in the structure of the surface layer. However, only a few works reported in the scientific literature mention/investigate the properties of the surface layer [19,20,21,22]. Jinkee Hong et al. [19] investigated the effect of polystyrene particles with a diameter of 0.4 μm to 100 μm on the cytotoxicity of human dermal fibroblasts and mast cells [23]. In the study by Jinkee Hong et al., polystyrene (PSt) particles from Sigma Aldrich and Cospheric (USA) were studied as model particles. The authors note that PSt particles with a diameter of 460 nm and 1 μm are characterized by a weak negative zeta potential of −2.2 mV, while larger microspheres (3 μm, 40 μm, 100 μm) have a weak positive zeta potential of +1.2 mV. The researchers believe that small particles at pH = 7 are characterized by a lower charge according to the DLVO theory. This statement seems controversial since the charge of the particle surface is determined primarily by the nature and concentration of surface functional groups and not by their size [24,25,26]. However, the presence of any functional group in the surface layer of particles is not mentioned in the work of Jinkee Hong et al. In the course of their study, Jinkee Hong showed that PSt particles with a diameter of 3 to 100 μm were not toxic to human dermal fibroblast cells and mast cells at an experimental dosage of 500 μg/mL and did not cause an allergic reaction or histamine-mediated inflammation. However, small PSt particles with a diameter of 460 nm and 1 μm affected erythrocytes, which caused their hemolysis. The study did not clarify why small PS particles caused the destruction of red blood cells, since ion–ion interactions between negatively charged red blood cells and PSt particles are excluded. The authors suggest that van der Waals interactions may exist between red blood cells and PSt particles, but the experimental data are not discussed in detail. In addition, it is shown that small PSt particles can cause local inflammation in tissues at a concentration of 500 μg/mL. It should be noted that the authors only considered the effect of size on the cytotoxicity of PS particles, while the effect of the surface layer structure on interactions with both blood cells and fibroblast cells was not taken into account.

Wei Xu et al. also conducted model studies on the penetration of PSt nanoparticles through the epidermal layers of human skin cells [27]. PSt nanoparticles from Sigma Aldrich with a diameter of 100 and 500 nm were studied, and the concentration of polystyrene particles was 1 ppm, which is 500 times lower than in the studies conducted by Jinkee Hong. The authors note that the surface layer of PSt nanoparticles contains carboxyl groups, and the modification of particles with a fluorescent label led to an insignificant increase in zeta potential (from −40 mV to −45–58 mV). The study noted that under simulated conditions, when there are no keratinized layers on the skin surface [28], penetration of nanoparticles into epidermal cells was observed, which forced keratinocytes to produce a greater number of inflammatory factors, components of the immune response, and signaling molecules. Wei Xu noted that the PSt nanoparticles studied were model particles and cannot be fully considered as nanoplastics found in nature. It is known that the surface layer of “real” microplastics contains various compounds sorbed from the environment—aromatic pollutants, surfactants, biologically active compounds, heavy atom ions, etc. [29,30].

Christian Laforsch studied PSt particles of 3 μm in diameter with no functional groups on the surface layer purchased from Micromod (Germany). He showed that the process of internalization by macrophages (absorption) of both modified PSt particles and the original ones depended on the presence of biologically active compounds in the surface layer of the microspheres. As a model experiment, Christian Laforsch kept the original PSt particles in fresh water from an artificial outdoor pool and in salt water (salinity 35‰) taken from an aquarium for 2 or 4 weeks [31]. The original PSt microspheres showed an absorption rate by macrophages that was 10 times worse than microspheres kept in different types of water, while no difference was observed between the types of water in which the particles were kept. However, the authors did not study the chemical composition of the water, only indicating that in the presence of water (from an aquarium or a pool), an “eco” crown is formed on the surface of polystyrene microspheres. In addition, the work also describes a model experiment in which IgG antibodies from mouse serum are covalently attached to polystyrene microspheres. Localization of immunoglobulin on the surface layer of polystyrene particles increased their internalization by macrophages 100-fold compared to the original polystyrene microspheres. Thus, it is obvious that the interaction of polymer model particles with cells of various natures or formed elements of blood will be affected by parameters such as particle size and the structure of the surface layer (zeta potential, the presence of functional groups, adsorbed biologically active substances, etc.).

Since researchers mostly use polystyrene particles that are spherical in shape, the question of whether the shape of polystyrene particles affects their sorption to various compounds present in the environment remains open. There are studies in the literature describing the influence of the shape of polystyrene particles on sedimentation and aggregation processes [32,33]. Modeling of the aggregation processes of polymer particles has shown that spherical particles form more stable aggregates. The need to develop a general approach to the use and study of polystyrene particles as model particles is beyond doubt. It is obvious that it is necessary not only to indicate the size of the polystyrene particles used in studies but also to describe the properties of the surface layer (the nature and concentration of functional groups, sorbed surface active compounds or biologically active compounds, the presence of a developed specific surface, etc.).

It is known that microplastics found in nature can contain various chemical pollutants in the surface layer: bisphenol A, phthalates, formaldehyde, benzene, triclosan, etc. [34]. Studying the sorption processes of these compounds in laboratory conditions does not always seem appropriate, since all of the listed compounds are toxic and can cause a number of diseases. Therefore, various organic luminophores are often considered as model compounds. On the one hand, it makes it possible to study the sorption process using spectrophotometry and optical luminescence microscopy [35]. On the other hand, organic luminophores, such as rhodamine, are often used in the agricultural industry [36]; thus, these aminoxanthene dyes enter the environment and can be adsorbed on microplastics.

In this work, we present the first results of a model experiment to study the effect of the shape and structure of the surface layer of polystyrene microspheres and non-spherical particles with sizes from 2 to 5 μm on the sorption of rhodamine B. The approach used in this study with model polystyrene microplastics and model pollutants will allow a better understanding of the interaction of microplastics with environmental matrices and with living bodies. The analysis of the effects not only of particle shape and dimension but also of their surface structure will allow the consideration of more real conditions of microplastics’ fates. Specifically, our model approach addresses our understanding of the behavior of primary and secondary microplastics subjected to long environmental degradation processes that will affect their surface and interactions with organic pollutants. The results of this study highlight the necessity of taking into account not only the size but also the structure of the surface layer of microplastic particles when studying the interaction of microplastics with other chemical compounds in the environment.

## 2. Materials and Methods

### 2.1. Materials

Polystyrene particles with diameters from 2 to 5 μm and of various shapes were synthesized using previously described methods [37,38,39]. It should be noted that all the studied particles were obtained in the presence of a crosslinking agent, divinylbenzene, which ensured the crosslinking of polystyrene chains and the fixation of the shape of the particles when kept in an organic solvent. The concentration of the crosslinking agent in the reaction mixture in all cases did not exceed 2 wt.%.

**Figure 1:**
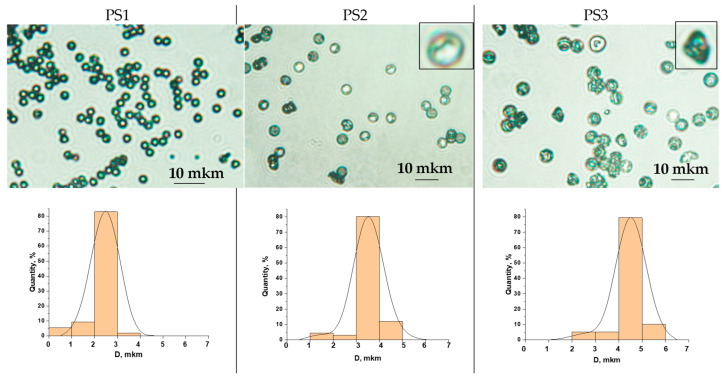
Optical microscopy photographs of model particles PS1, PS2, and PS3, as well as their size distributions. The inset shows an enlarged image of model particles PS2 and PS3. (For more detailed optical microscopy data, see SI Figure S1).

Table 1 and Figure 1 show the main characteristics of the polystyrene particles studied in this work. R-grade N,N-dimethylformamide (DMF), ethanol, and rhodamine B were purchased from Vekton LLC (Saint-Petersburg, Russia) and used without additional purification.

### 2.2. Modification of the Surface Layer of Polystyrene Particles

The structure of the surface layer of polystyrene particles was modified by placing the initial PS particles in DMF. For this purpose, an aqueous dispersion of polystyrene particles was dried to a constant weight. A 0.2 g sample was placed in a 5 mL test tube, 2 mL of DMF was added, and the mixture was left to stir on a shaker for 48 or 96 h. After the specified time, the particles were centrifuged (Eppendorf 5424 centrifuge, Hamburg, Germany), the supernatant liquid was decanted, and the particles were washed with ethanol 3–4 times. Then, the particles were dried at a temperature of 298.15 K and weighed (OHAUS Pioneer PR224 analytical balance, Nanikon, Switzerland). In addition, the content of washed-out oligomeric polystyrene chains was determined in the supernatant liquid after drying the solvent.

### 2.3. Determination of the Surface Area, Average Pore Diameter, and Pore Volume of Polymer Particles

The surface area, average pore diameter, and pore volume of polymer particles were determined by physisorption analysis (N_2_ and 77 K). The surface area, diameter, and volume of pores were calculated by observing the adsorption of a gas on the particles [40]. The Quadrasorb SI analyzer by Quantachrome Instruments (Boynton Beach, FL, USA) was used for physisorption on polymer microspheres; the surface area was calculated using the Brunauer–Emmett–Teller (BET) method, and pore size distribution with the Barrett–Joyner–Halenda (BJH) method. Preparation of samples consisted of drying polymer particles at 298.15 K for 24 h.

### 2.4. Study of Rhodamine B Sorption

Sorption of rhodamine B was carried out both on the initial PS particles and on the particles after their treatment with DMF. First, 1 mL of ethanol was added to a weighed portion of particles (30 mg), and the mixture was redispersed in an ultrasonic bath (Sapphire 2.8, Moscow, Russia) for 10–15 min. Then, 10 mL of NaCl solution (10^−3^ M) was added to the particles and they were treated with ultrasound in an ultrasonic bath for 10–15 min. A solution of rhodamine B in ethanol with a concentration of 0.24 g/L was preliminarily prepared. The rhodamine B solution was added to the water–salt dispersion of PS particles in the concentration range from 0 to 5 mg/mL. The sorption process was continued for 2 h, after which the particles were centrifuged at 10,000 rpm for 10 min in a centrifuge (Eppendorf 5424, Hamburg, Germany). The content of rhodamine B in the supernatant was determined using a pre-built calibration curve on a spectrophotometer (UV−1700 Plus, Shimadzu, Kyoto, Japan) at a wavelength of 550 nm.

### 2.5. Characterization by Optical Microscopy, Transmission Electron Microscopy (TEM), and Atomic Force Microscopy (AFM)

The structure of the surface layer and the size of both the initial PS particles and after their soaking in DMF were studied by optical microscopy (Olympus BX51 optical microscope, Tokyo, Japan) and transmission electron microscopy (TEM) (Zeiss Libra 200fe microscope, Oberkochen, Germany). Before TEM measurements, an aqueous dispersion of PS particles was applied to molding substrates treated with a 1% sodium dodecyl sulfate solution. Photographing was performed after the substrate had completely dried. Atomic force microscopy (AFM) images were captured with Veeco diNanoForce V (New York, NY, USA) operating in tapping mode (TM). AFM probe NT-MDT HA_NC Etalon (Moscow, Russia), resonant frequency 137 kHz force constant 3.5 N, and scan rate of 0.3 Hz (15 mkm/s for 25 × 25 mkm). Images were processed with Veeco Nanoscope Analysys 1.2 software. The size distributions of the microspheres, as well as their coefficients of variation (CV, defined as the standard deviation in the measured diameter divided by the average diameter), were calculated from the measured value of the produced particles.

### 2.6. Analysis of Experimental Data on the Sorption Capacity of Rhodamine B

The analysis of experimental data on the sorption capacity with respect to rhodamine B was carried out using the Langmuir model. For this purpose, linear isotherms were constructed and the parameters were calculated using Formula (1).

(1) $$\frac{1}{q}=\frac{1}{C_{e}\cdot K_{L}}\cdot \frac{1}{q_{max}}+\frac{1}{q_{max}}$$

where q is the adsorption value, mol/g; q_max_ is the maximum adsorption value, mol/g; K_L_ is the Langmuir constant, g/mol; and C_e_ is the equilibrium concentration, mol/g.

The applicability of the theoretical model of adsorption isotherms to the obtained experimental data was assessed based on the determination coefficient (R^2^) calculated in Origin 2019 software.

Statistics: The experiments were performed with *n* = 3–4. All data measurements were represented as means ± standard deviations.

## 3. Results

### 3.1. Characteristics of the Initial Model PS Particles

**Scheme 1:**
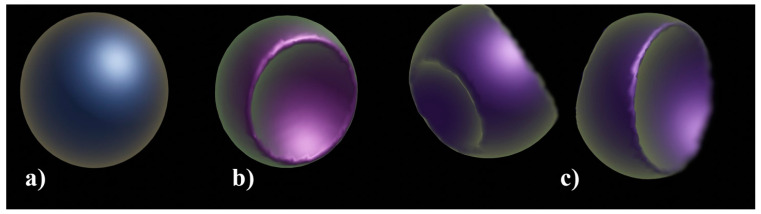
The shape of the model PS particles: spherical (**a**), with one dent (**b**), and non-spherical (biconcave discs) (**c**).

In this work, model cross-linked PS particles of various shapes were investigated. Scheme 1 shows a schematic representation of the particles investigated in this work. PS1 (Scheme 1a) shows microspheres, PS2 (Scheme 1b) is characterized by the presence of one dent, and PS3 (Scheme 1c) represents non-spherical particles.

Table 1 shows the data on the specific surface area of the initial PS microspheres. Despite the fact that the shape of the particles is different, the values of the measured specific surface area lie in a narrow range from 3 to 3.3 m^2^/g. The theoretical calculation of the specific surface area based on the shape of the particles shows that the values should be significantly lower (Table 1).

**Figure 2:**
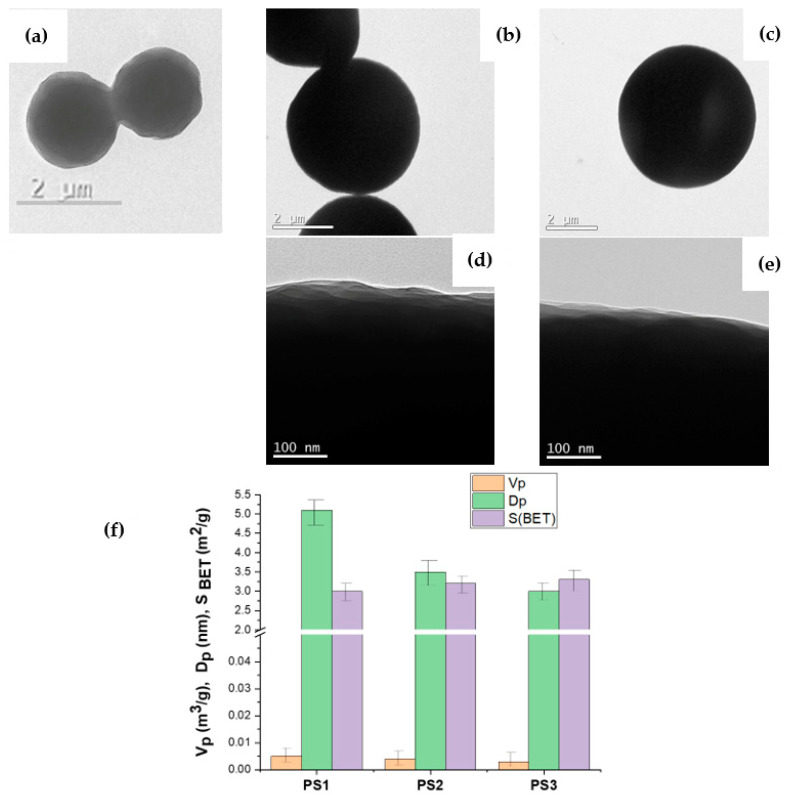
TEM images of PS particles: PS1 (**a**), PS2 (**b**), and PS3 (**c**). Structure of the surface layer of PS2 (**d**) and PS3 (**e**) particles. Characteristics of the particles (**f**): BET analysis: pore volume (Vp), average pore size (Dp), and specific surface area (S_BET_).

The difference between the theoretical calculations and the experimental data when measuring the specific surface area indicates that the structure of the surface layer of the particles is not uniform and smooth. These conclusions are confirmed by the data obtained in the study of the surface structure of the particles using transmission electron microscopy (Figure 2).

As seen in Figure 2d,e, the structure of the surface layer of all model particles is not smooth and non-uniform. In addition, the increase in the specific surface area of the PS particles can also be associated with the presence of micro- and mesopores. However, as evidenced by the data obtained after measuring the pore volumes and their size distribution (Figure 2f), the pore volume values for all the studied microspheres are comparable and do not exceed 0.005 m^3^/g, while micropores are absent in the initial PS microspheres; only pores with a size of 5 to 15 μm are detected in the surface layer.

**Figure 3:**
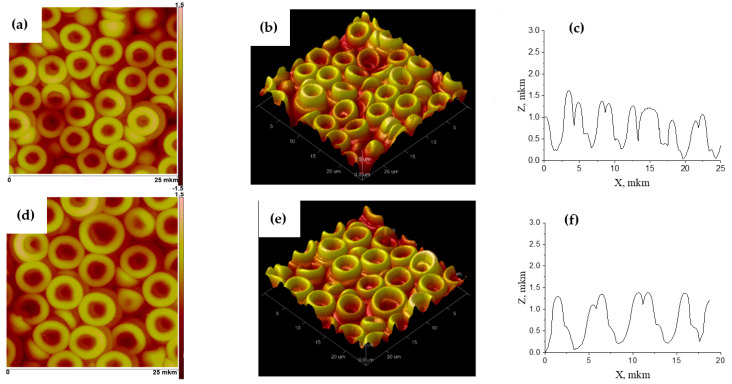
AFM images of PS2 (**a**–**c**) and PS3 (**d**–**f**) model particles.

The study of the surface structure of the initial PS particles using the AFM method indicates that the PS2 and PS3 samples are characterized by the presence of dents, while the depth of the dent is 1.0 and 1.2 μm, respectively (Figure 3).

Thus, model cross-linked polystyrene particles of different shapes (spherical, with one dent, and non-spherical biconcave) were selected for the study. The structures of the surface layer of such particles are very similar (comparable values of specific surface area and pore volume) and, obviously, the polymer chains are tightly packed in the surface layer, while the content of mesopores is minimal.

### 3.2. Modification of the Structure of the Surface Layer of Model PS Particles

**Figure 4:**
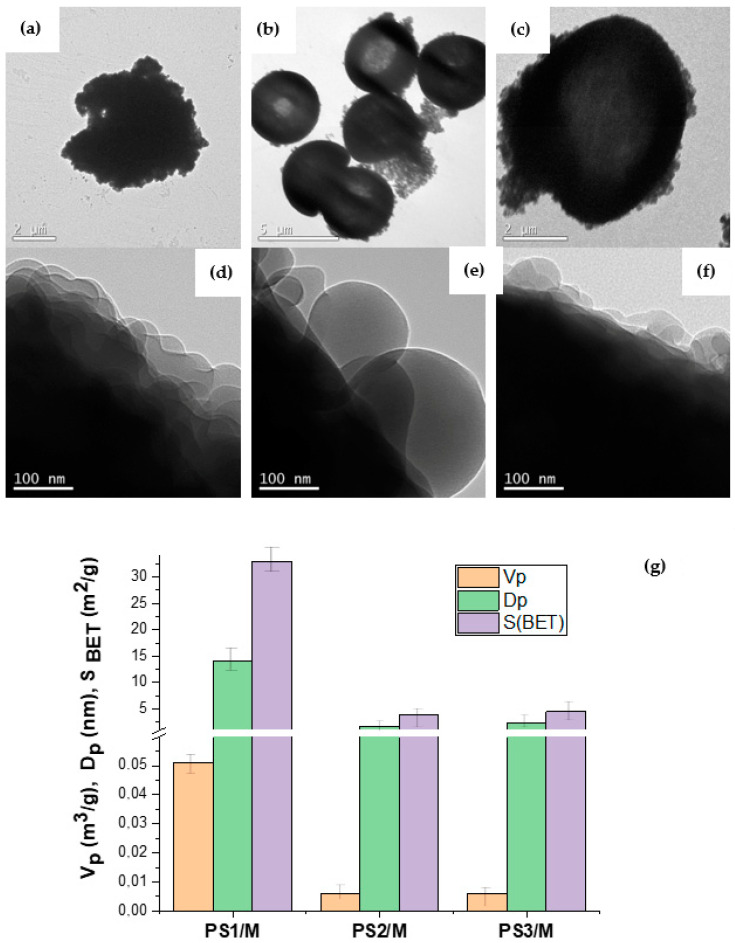
TEM images of modified PS particles: PS1/M (**a**), PS2/M (**b**), and PS3/M (**c**). The structure of the surface layer of PS1/M (**d**), PS2/M (**e**), and PS3/M (**f**) particles. Characteristics of the particles (**g**): BET analysis: pore volume (Vp), average pore size (D_p_), and specific surface area (S_BET_).

N,N′-dimethylformamide (DMF) was used as an organic solvent capable of modifying the surface of model polystyrene microspheres. DMF is a common organic solvent and is used in many industries, including plastics production and pharmaceuticals [41]. When released into the environment, DMF can be sorbed by microplastic particles, causing changes in the structure of the surface layer. Meanwhile, DMF does not change the chemical structure of polystyrene-based plastic [42]. In this work, the effect of DMF exposure time on the properties of the surface layer of model PS particles was traced. It was shown that regardless of the exposure time (48 or 96 h), the presence of a DMF solvent results in the diffusion of non-crosslinked polystyrene chains from the surface layer or the volume of the particles into the dispersion medium. As a consequence, the polymer chains in the particles are packed “less” densely, as clearly seen in the TEM images (Figure 4).

Furthermore, the shape of PS1 microspheres after immersion in DMF for 96 h was no longer spherical (Figure 4a), and the structure of the surface layer became “raspberry-like” (Figure 4d), which indicates the diffusion of polymer chains from the bulk of the microspheres to the surface. As follows from the TEM images, the mobility of polymer chains in the surface layer of the model particles is different. Thus, the most labile polymer chains are characteristic of the PS2/M particle (Figure 4e); the formation of spherical “growths” of 70 to 150 nm in size is detected on the surface of such particles due to the extension of polymer chains. In addition, by removing DMF from the supernatant and weighing the dry residue, it was found that non-crosslinked polymer chains diffused from the bulk of model PS1/M, PS2/M, and PS3/M particles during the first 48 h in amounts of 7.1 wt.%, 3.3 wt.%, and 3.1 wt.%, respectively. Increasing the immersion time of the model polystyrene particles in DMF up to 96 h leads to the diffusion of 25 wt.%, 18 wt.%, and 19 wt.% of the polystyrene chains from the bulk of the PS1/M, PS2/M, and PS3/M particles, respectively.

As we have shown previously [43], in the process of the emulsion polymerization of styrene, the composition of microspheres can include oligomeric chains with a molecular weight of up to 10,000 while the main proportion of polymer chains will be characterized by a molecular weight of 100,000 to 500,000. According to the scientific literature [42], polymer chains of polystyrene with an average molecular weight not exceeding 54,000 can be dissolved in DMF. Thus, the use of DMF as a solvent makes it possible to remove oligomeric chains from the structure of model polystyrene particles. The study of the specific surface area and pore volume of model polystyrene particles after their modification in DMF showed that PS1/M particles are characterized by a significant increase in the specific surface area of up to 33 m^2^/g (an increase of 11 times) due to the loss of their spherical shape and the maximum amount of non-crosslinked polystyrene chains being washed out. At the same time, PS2/M and PS3/M particles are characterized by a less significant increase in the specific surface area after their exposure to DMF—up to 3.9 and 4.5 m^2^/g, respectively (1.2- and 1.3-fold increase). The increase in the specific surface area of the modified particles occurs not only due to a change in the surface structure, as shown in the TEM micrographs (Figure 4), but also due to the additional formation of micro- and mesopores. After 96 h in DMF, PS1/M particles are mainly characterized by the presence of mesopores of 3 to 20 nm, with the total volume of these pores being 93%. After 96 h in DMF, PS2/M and PS3/M particles are mainly characterized by the presence of micropores with sizes of 1.7 to 2.5 nm and a total volume of 83%. Thus, the analysis of the structure of the surface layer and the pore structure of the model particles after their modification in DMF showed that even after a short time (4 days) of keeping the polymer polystyrene particles in the DMF medium, the morphology of the microspheres significantly changed. In addition, it should be noted that not only does the morphology of the particles change but the hydrophobic properties of their surface layer are also altered. The initial polystyrene particles, regardless of their shape, can be easily dried and redispersed back into an aqueous medium using an ultrasonic bath. However, after removing DMF-soluble polystyrene chains (with an average molecular weight of less than 54,000) from the surface layer, the structure of the surface layer of the modified microspheres becomes more hydrophobic, which is manifested in the poor wetting of their surface with water. As a result, even prolonged exposure to ultrasound (more than 15–20 min) does not allow redispersion of such particles in an aqueous medium. Nevertheless, the preliminary addition of 1–2 mL of ethyl alcohol to dry particles leads to good wettability of the surface of the modified particles and their subsequent redispersion in an aqueous or water–salt dispersion medium.

Thus, this study showed that spherical cross-linked polystyrene microspheres undergo a significant change in shape in the presence of DMF, while no significant change of shape is observed for non-spherical particles. In addition, keeping polystyrene particles in DMF increases the hydrophobicity of their surface layer and leads to the formation of additional porosity in the surface layer.

### 3.3. Study of Rhodamine B Adsorption on Model PS Particles

**Figure 5:**
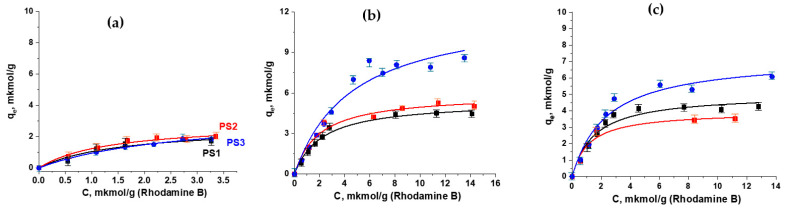
Adsorption of rhodamine B by model polystyrene particles of different shapes: spherical (PS1), with one dent (PS2), and non-spherical biconcave (PS3). Adsorption on the initial model particles (**a**) and particles after modification in DMF for 48 h (**b**) and 96 h (**c**). The Langmuir model is applied for the adsorption isotherms of rhodamine B. Points are experimental data, and lines are model data.

The study of the sorption of rhodamine B molecules on both the initial model polystyrene particles and the modified ones was carried out in a water–ethanol medium containing a background concentration of NaCl salt (10^−3^ M). It turned out that, for the initial polystyrene particles, the sorption capacity does not depend on the shape of the particles (Figure 5a). The maximum experimental sorption capacity for all particles lies in a narrow range from 1.7 to 2.0 μmol/g. At the same time, spherical PS1 microspheres adsorb 15% less rhodamine B compared to non-spherical PS2 and PS3 particles, which is apparently due to the fact that non-spherical particles are characterized by slightly larger specific surface areas (Table 1). After keeping the polystyrene particles in a DMF medium for 48 h, their sorption capacity increased significantly: by 2.6, 2.3, and 4.7 times for PS1/M, PS2/M, and PS3/M, respectively. Modification of the structure of the surface layer of the particles for 96 h in a DMF medium led to the sorption capacity of rhodamine B being 4.3, 3.5, and 6.1 μmol/g for PS1/M, PS2/M, and PS3/M, respectively.

As can be seen, the sorption capacity of model spherical PS1 microspheres does not depend on the modification time in DMF, while the sorption capacity of non-spherical particles depends on the time of solvent exposure. A 30% decrease in the sorption capacity for non-spherical particles after 2 days of DMF exposure may be associated with the reconstruction of the surface layer with the increase in the solvent exposure time. For a better understanding of the mechanism of the sorption process, the Langmuir model was used (Table 2).

The calculated maximum values of the sorption capacity are 10–18% higher than the experimental values (Table 2). The Langmuir model assumes the formation of a monolayer on the adsorbed surface. Thus, it is obvious that the experimental values do not reach the possible maximum theoretical values.

The obtained determination coefficients for the Langmuir model showed high values (0.94–0.98) for all the studied particles, which confirms that this model describes the obtained experimental data well. Analysis of the sorption capacity values of the original polystyrene and modified particles shows that an increase in the specific surface area and the degree of hydrophobicity of the particles leads to a significant increase in the adsorption of rhodamine B. However, at this stage of the work, we were unable to trace the relationship between the effects of pore volume and pore size on the adsorption of rhodamine B. According to the literature [44], when the particle size is smaller, the sorption capacity is higher due to the increase in the specific surface area and the number of active centers responsible for the sorption processes. Obviously, the sorption of rhodamine B on modified particles is determined not solely by the volume and size of the pores. In our experiments, the maximum increase in the specific surface area and pore volume is observed for PS1/M particles; however, the sorption capacity of such microspheres is insignificant. These particles are mainly characterized by the formation of mesopores (from 5 to 20 nm), and since monomolecular adsorption of rhodamine B is observed, some of the pore volume is unoccupied by adsorbate molecules. In [45], it is shown that the adsorption of rhodamine B on PVC, PS, and PET microplastic particles depends on the values of the specific surface area, hydrophobicity, and crystallinity of the polymer. The authors conclude that a decrease in hydrophobicity, an increase in the specific surface area, and the presence of an amorphous structure contribute to the maximum adsorption of the organic pollutant—rhodamine B. However, in our work, we studied only polystyrene-based particles, and, during their modification, only the hydrophobicity of the surface layer of the particles was increased. Therefore, for PSt particles, hydrophobic interactions are one of the main conditions determining the process of surface adsorption of rhodamine B molecules. For a more detailed understanding of the adsorption processes, FTIR spectra were recorded for both the initial particles and after their modification in a DMF medium, as well as after the adsorption of a model pollutant, rhodamine B (see Supporting Information Figures S3–S8). It is shown that there are no changes in the chemical structure of the initial polystyrene particles after their modification by DMF, and changes in the chemical structure after adsorption of rhodamine B are also observed. Thus, no additional functional groups appeared in the structure of the polymer chains after modification of the model polystyrene particles in the experimental conditions under study. The adsorption process of the model pollutant is most likely carried out by hydrophobic and π–π interactions. In addition, it turned out that a significant increase in the specific surface is a necessary but not sufficient condition for increasing the sorption capacity. Obviously, further studies are needed to understand the processes occurring in the surface layer of polymer particles in the presence of various solvents, and it is also necessary to identify the factors and patterns that determine the processes of sorption of various organic substances on the surface, which have undergone significant changes under the influence of various solvents.

## 4. Discussion

This study addresses two main issues. In the first part of our study, we decided to answer the question of whether the shape of polystyrene particles (spherical, with one dent, or non-spherical biconcave) affects the sorption process of the aromatic dye rhodamine B. We showed that regardless of the shape of the polystyrene model particles used in this study, the values of the sorption capacity were comparable. Thus, if we use model polystyrene particles with a uniformly structured surface layer (comparable values of specific surface, degree of hydrophobicity, etc.) for this study, we can choose spherical particles for simplicity. The synthesis of such spherical particles is a one-stage process and is well-studied. Therefore, there is no need to develop new methods for the synthesis of model polystyrene particles of complex morphology. However, in the second part of our work, we tried to answer the question of what happens when the model polystyrene particles are exposed to an organic solvent, using DMF as an example. It turned out that spherical particles undergo significant changes not only in shape but also in the structure of the surface layer. At the same time, such changes for non-spherical particles were not revealed during the experimental work. It is obvious that various types of polymer particles are present in the environment. A large number of polymer polystyrene particles are synthesized and used in various aspects of our lives: for the calibration of devices, as carriers in the diagnosis of various diseases, etc. The resulting particles can end up in the environment and affect it, so the choice of polystyrene particles as model particles is justified. However, our results confirm that it is less necessary to take into account the shape of the particles, and more so the properties of the surface layer. Studies on changes in the structure of the surface layer of polystyrene particles under the influence of the DMF solvent show that research in this direction should be continued since the adsorption processes are affected not only by the specific surface of the model particles but also by the pore size distribution, pore volume, hydrophobicity of polymer chains, and mobility in the surface layer, etc.

## 5. Conclusions

This paper considers model polystyrene particles of spherical and non-spherical shape. It is shown that when the structure of the surface layer of particles consists of densely packed polystyrene chains, the shape of the model particles does not affect the process of sorption of the model dye—rhodamine B. In addition, it is shown that for all microspheres before DMF treatment, the pore volume is comparable and does not exceed 0.005 m^3^/g, there are no micropores, and only pores from 5 to 15 microns in size are found in the surface layer. Varying the structure of the surface layer of particles in the process of removing oligomeric chains under the influence of the solvent made it possible to establish that a significant increase in the specific surface of the particles is a necessary but not sufficient condition determining the process of adsorption of rhodamine B molecules. The increase in the specific surface area of the modified particles occurs not only due to a change in the surface structure, as shown in the TEM micrographs, but also due to the additional formation of micro- and mesopores. It is obvious that the presence of micropores with a diameter of 1.7 to 2 nm has a significant effect on the sorption capacity. In addition, we believe that the sorption capacity can be affected by an increase in π–π interactions between dye molecules and the moiety polymer chains of modified microspheres, which is indirectly confirmed by an increase in hydrophobicity, which is manifested in the poor wetting of their surface with water. Further research in this area will allow us to understand and predict the processes occurring with polymeric polystyrene microplastics found in the environment.

## Figures and Tables

**Table 1:** Characteristics of the initial polystyrene particles.

Sample	D, mkm	CV ^1^	Surface Area (S), m^2^/g		Shape
			Theoretical	Experimental	
PS1	2–3	17	2.9–1.9	3.0	spherical
PS2	4	11	1.4	3.2	sphere with one dent
PS3	5	15	1.1	3.3	non-spherical

^1^ CV data are determined by calculation in ImageJ (version 1.53j).

**Table 2:** Main parameters of the Langmuir model of rhodamine B adsorption isotherms on the initial and modified polystyrene model particles.

Parameters of the Langmuir Model	PS1	PS1/M		PS2	PS2/M		PS3	PS3/M	
		48 h	96 h		48 h	96 h		48 h	96 h
q_exp_ μmol/g	1.71	4.4	4.3	2.02	5.0	3.5	1.84	8.6	6.1
q_max theory_, μmol/g	2.68	5.4	5.0	2.87	5.9	4.0	3.45	12.1	7.3
R^2^	0.94	0.98	0.95	0.97	0.98	0.96	0.99	0.95	0.96

## Data Availability

The original contributions presented in the study are included in the article/Supplementary Materials, further inquiries can be directed to the corresponding authors.

## Supplementary Materials

The following supporting information can be downloaded at: https://www.mdpi.com/article/10.3390/polym16223159/s1, Figure S1: video obtained with an optical microscope—movement of polystyrene particles of various shapes; Figure S2: TEM images of the surface layer structure of model polystyrene particles after their modification in a DMF medium; Figure S3: FTIR spectra of model polystyrene particles with one dent (PS2) and after adsorption of rhodamine B by initial particles (PS2 + rhod.B) and after modification in DMF for 48 h (PS2/4M + rhod.B) and 96 h (PS2/9M + rhod.B) (in the KBr pellet.); Figure S4: FTIR spectra of model polystyrene particles with one dent (PS3) and after adsorption of rhodamine B by initial particles (PS3 + rhod.B) and after modification in DMF for 48 h (PS3/4M + rhod.B) and 96 h (PS3/9M + rhod.B) (in the KBr pellet.); Figure S5: Original FTIR spectra of model polystyrene particles PS3; Figure S6: Original FTIR spectra of model polystyrene particles PS3 after adsorption of rhodamine B; Figure S7: Original FTIR spectra of model polystyrene particles PS3/4M + rhod.B; Figure S8: Original FTIR spectra of model polystyrene particles PS3/9M + rhod.B.
