# Supplementary material for: Effects of Particle Shape and Surface Structure on the Adsorption Properties of Polystyrene Microplastics
Source: Polymers (Basel). 2024 Nov 13;16(22):3159. doi: 10.3390/polym16223159 (PMC11598476; doi:10.3390/polym16223159)
Supplement: Supplementary file 1 [file polymers-16-03159-s001.zip › polymers-3274561-supplementary.pdf]

polymers

Supporting Information

Article

Effects of Particle Shape and Surface Structure on the Adsorption Properties of Polystyrene Microplastics

Natalia Shevchenko [1,*], Olga Iakobson [1,*], Vladimir Isakov [1] and Ivan Zorin [1,2]

[1] Microplastics Research Center, Yaroslav-the-Wise Novgorod State University; B. St. Petersburgskaya Str. 41, 173003 Veliky Novgorod, Russia
[2] Department of Chemistry, Saint Petersburg State University, University Av., 26, 198504 Saint Petersburg, Russia
* Correspondence: natali.shevchenko29@gmail.com (N.S.); iakobson.olga@yandex.ru (O.I.)

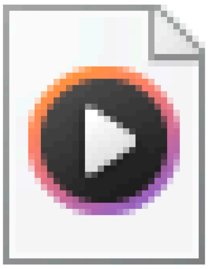

PS2.mp4

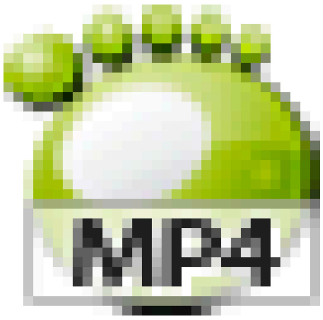

PS3.mp4

Figure S1. Video obtained with an optical microscope - movement of polystyrene particles of various shapes (PS2 and PS3 microspheres, properties are given in Table 1).

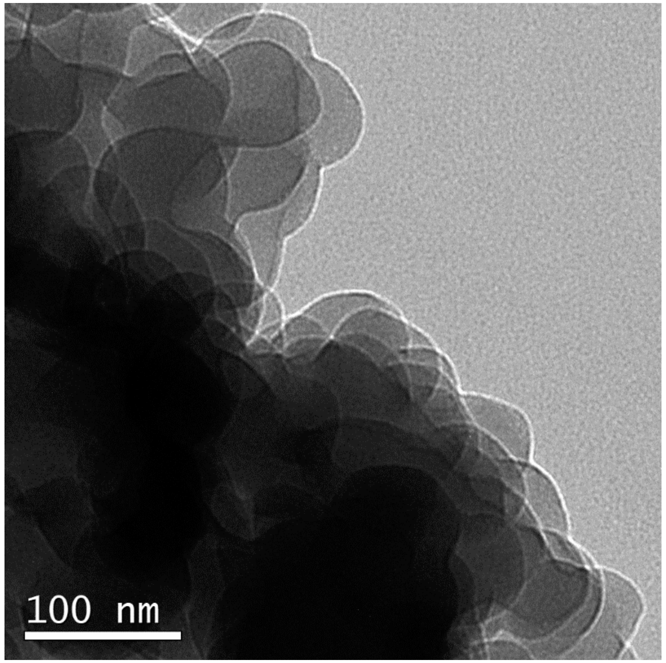

PS1

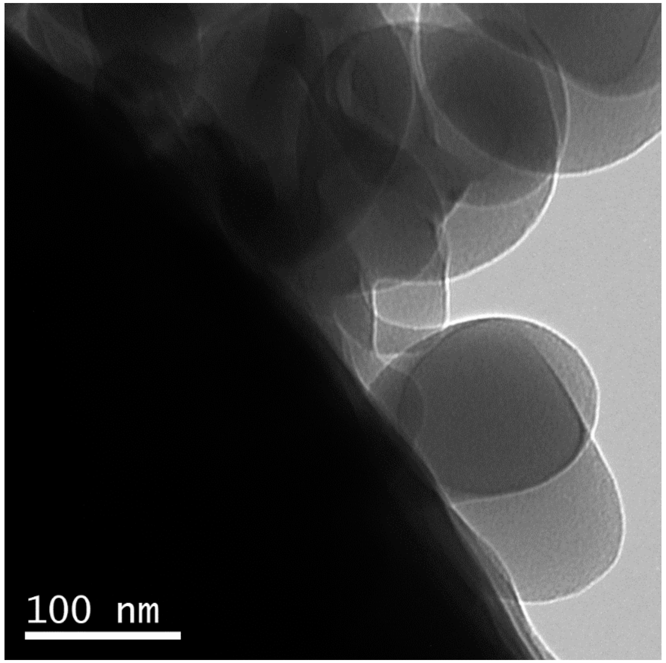

PS2

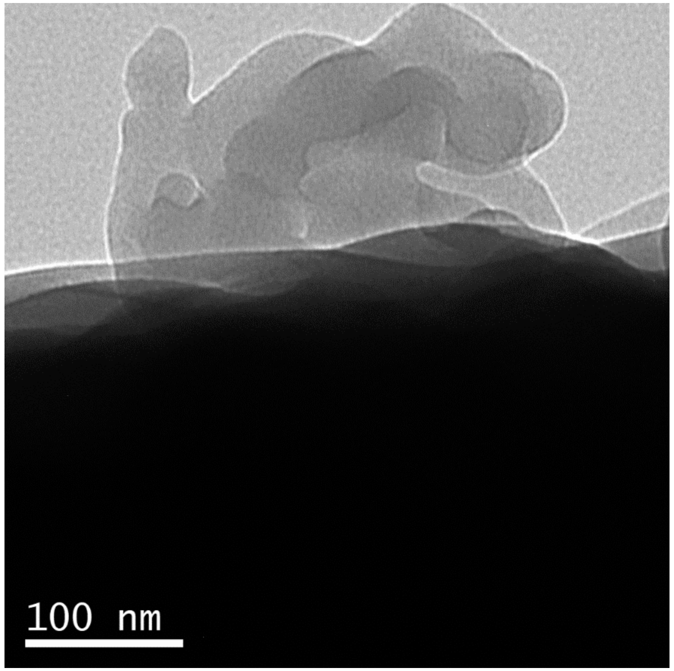

PS3

Figure S2: TEM images of the surface layer structure of model polystyrene particles after their modification in DMF medium.

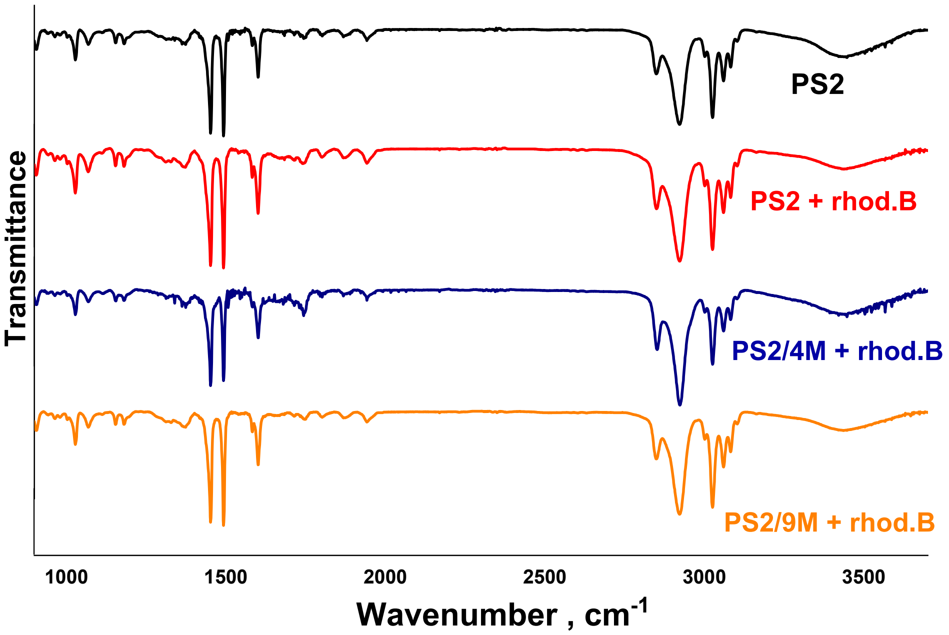

Figure S3: FTIR-spectra of model polystyrene particles with one dent before (PS2) and after adsorption of rhodamine B (PS2 + rhod.B); after keeping in DMF for 48 h with the following adsorption of rhodamine B (PS2/4M +rhod.B) or for 96 h with the following adsorption of rhodamine B (PS2/9M +rhod.B) (in the KBr pellet).

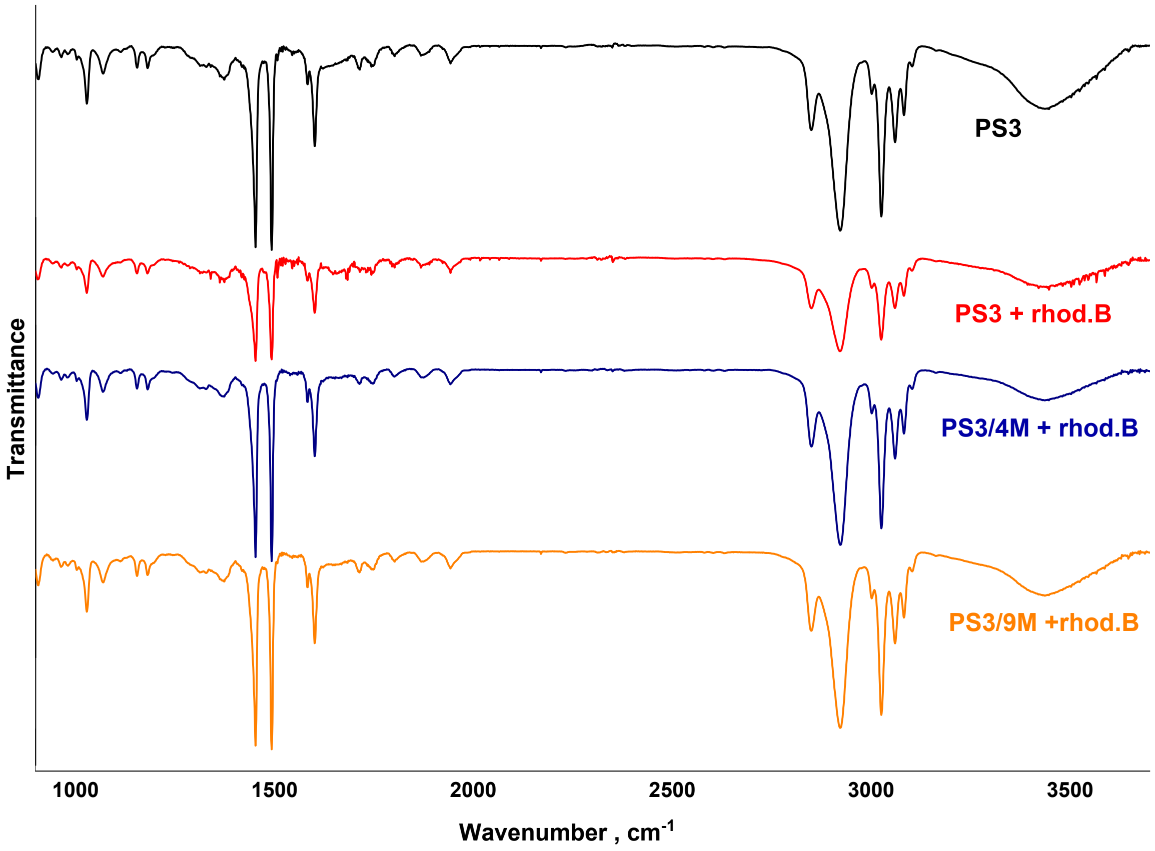

Figure S4. FTIR-spectra of model polystyrene particles with one dent before (PS3) and after adsorption of rhodamine B (PS3 + rhod.B); after keeping in DMF for 48 h with the following adsorption of rhodamine B (PS3/4M +rhod.B) or for 96 h with the following adsorption of rhodamine B (PS3/9M +rhod.B) (in the KBr pellet).

The chemical structure of model polystyrene particles before and after adsorption of rhodamine B in KBr pellets were studied by FTIR spectroscopy (IRPrestige-21, Shimadzu). All the spectra were recorded in the range of 400-4500 cm^{-1} and represented an average of 60 scans taken. It is shown that there are no changes in the chemical structure of the initial polystyrene particles after their modification by DMF, and the changes in the chemical structure after adsorption of rhodamine B are observed also.

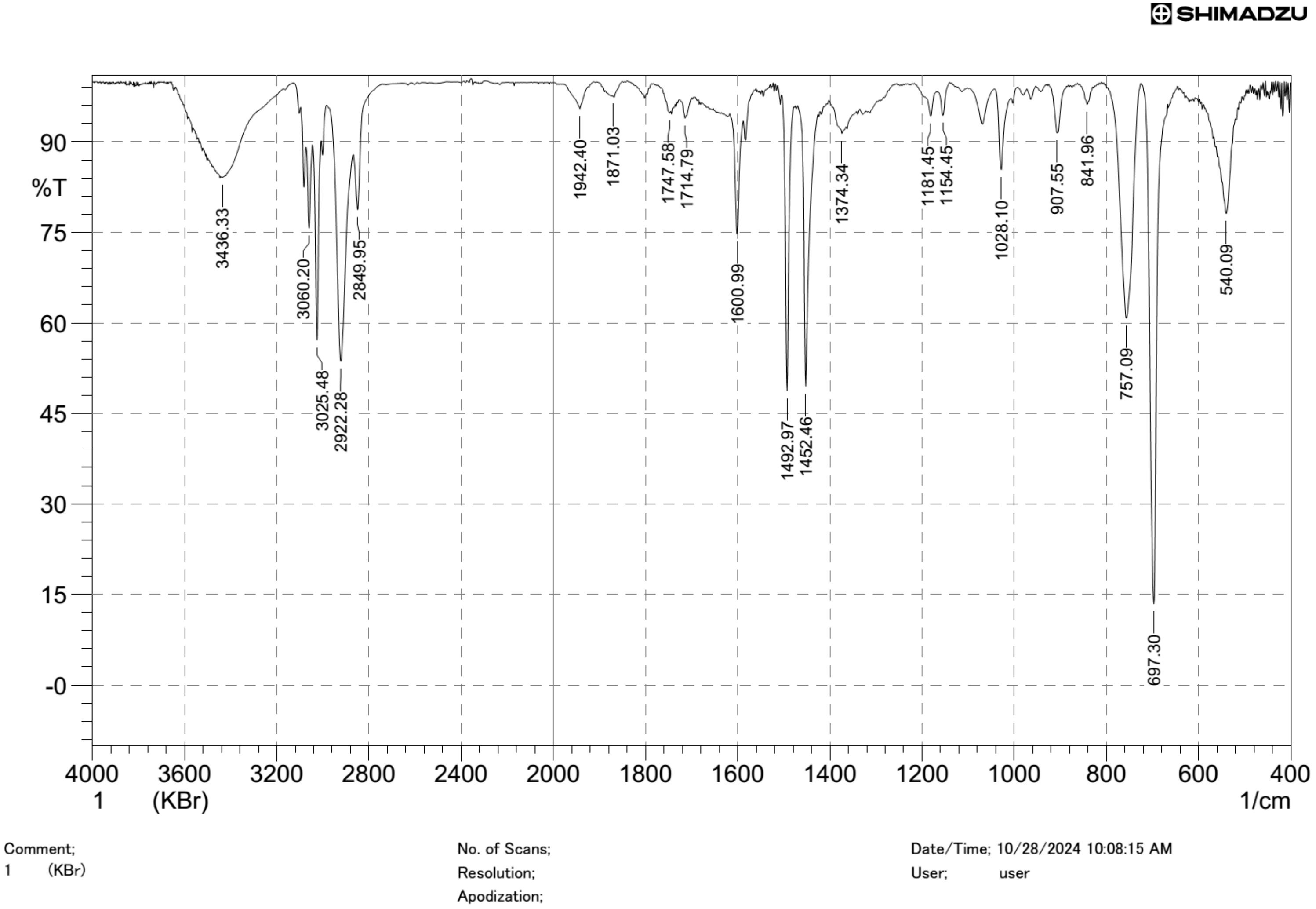

Figure S5: Original FTIR-spectra of model polystyrene particles PS3

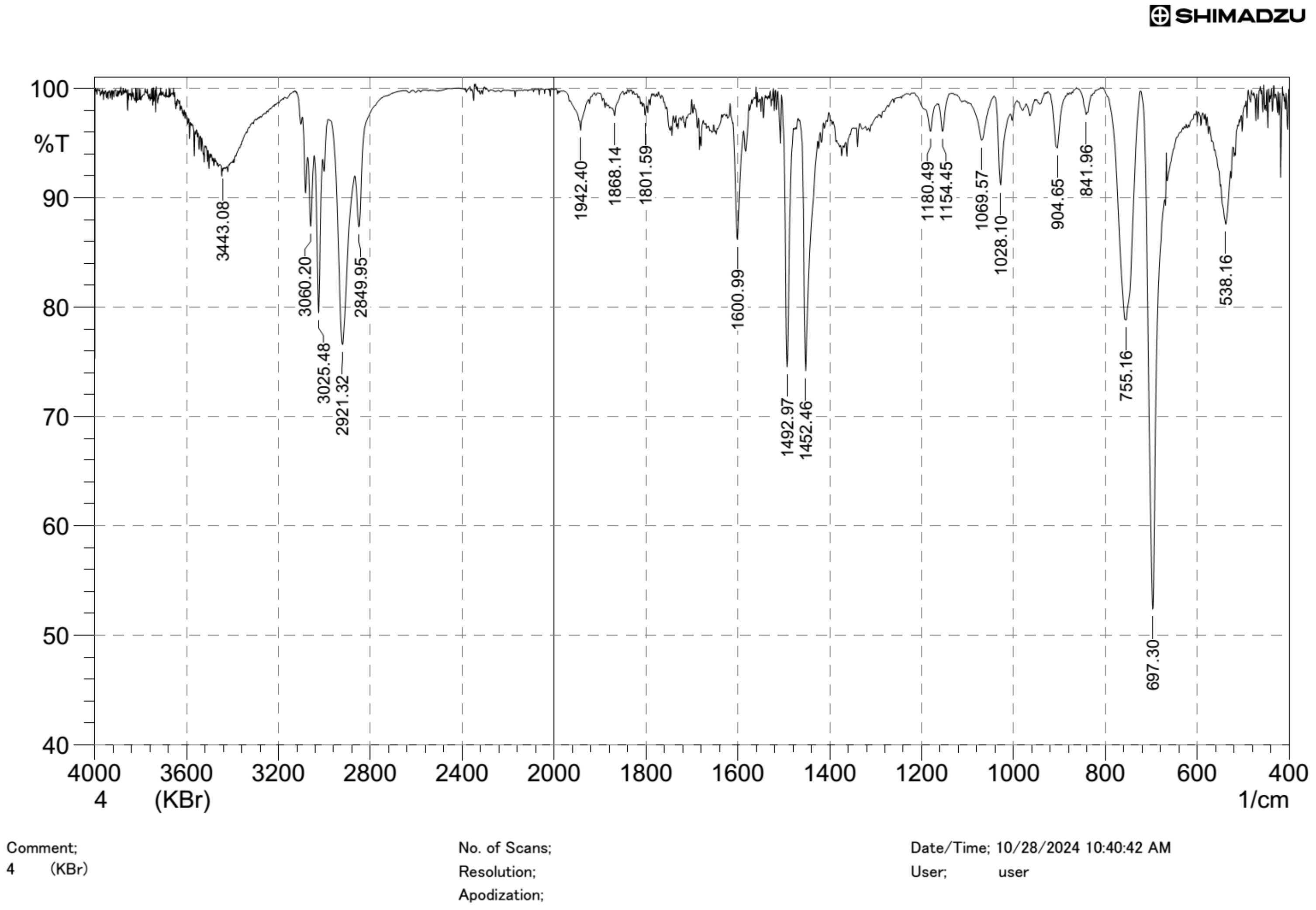

Figure S6: Original FTIR-spectra of model polystyrene particles PS3 after adsorption of rhodamine B

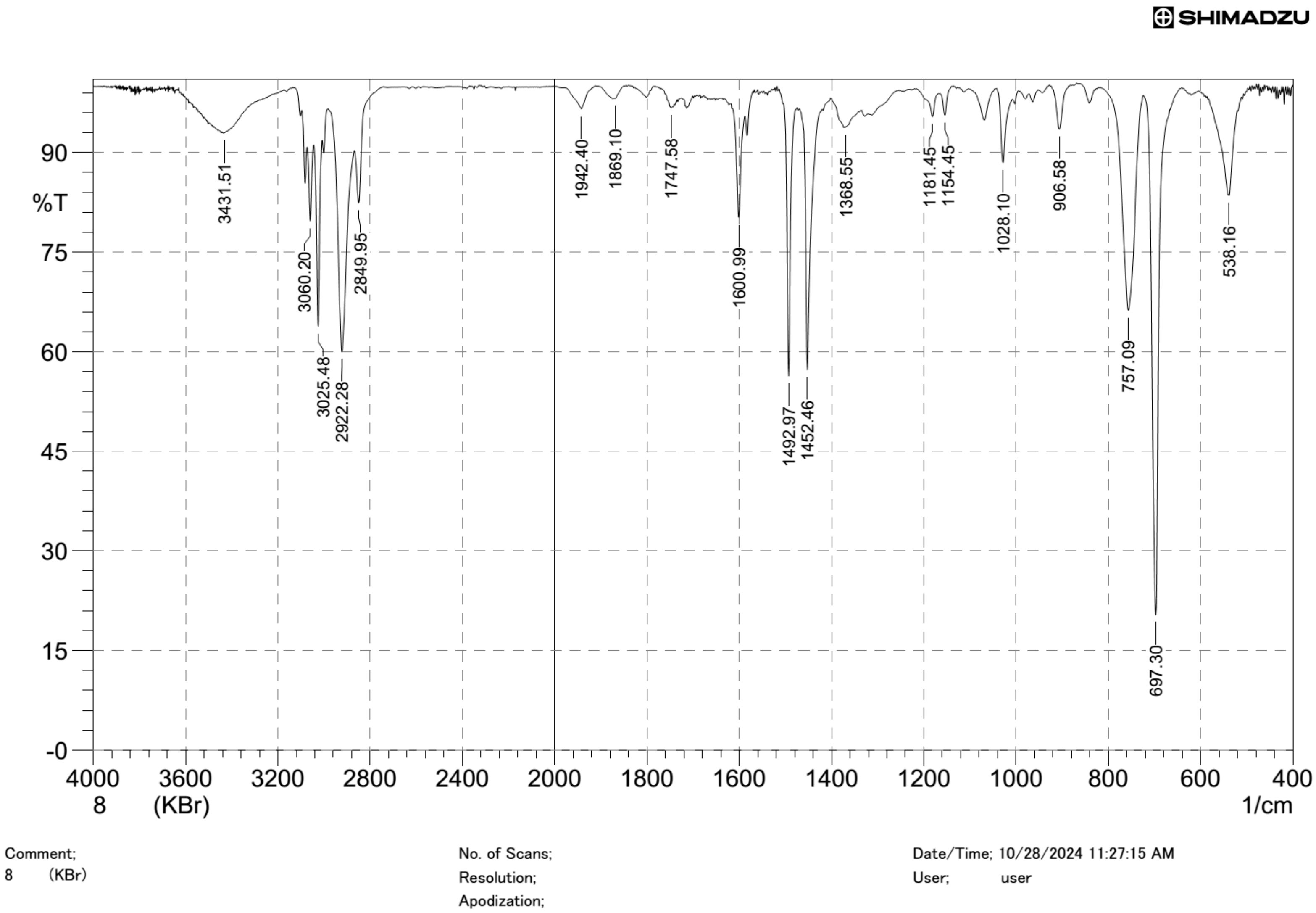

Figure S7: Original FTIR-spectra of model polystyrene particles PS3/4M +rhod.B

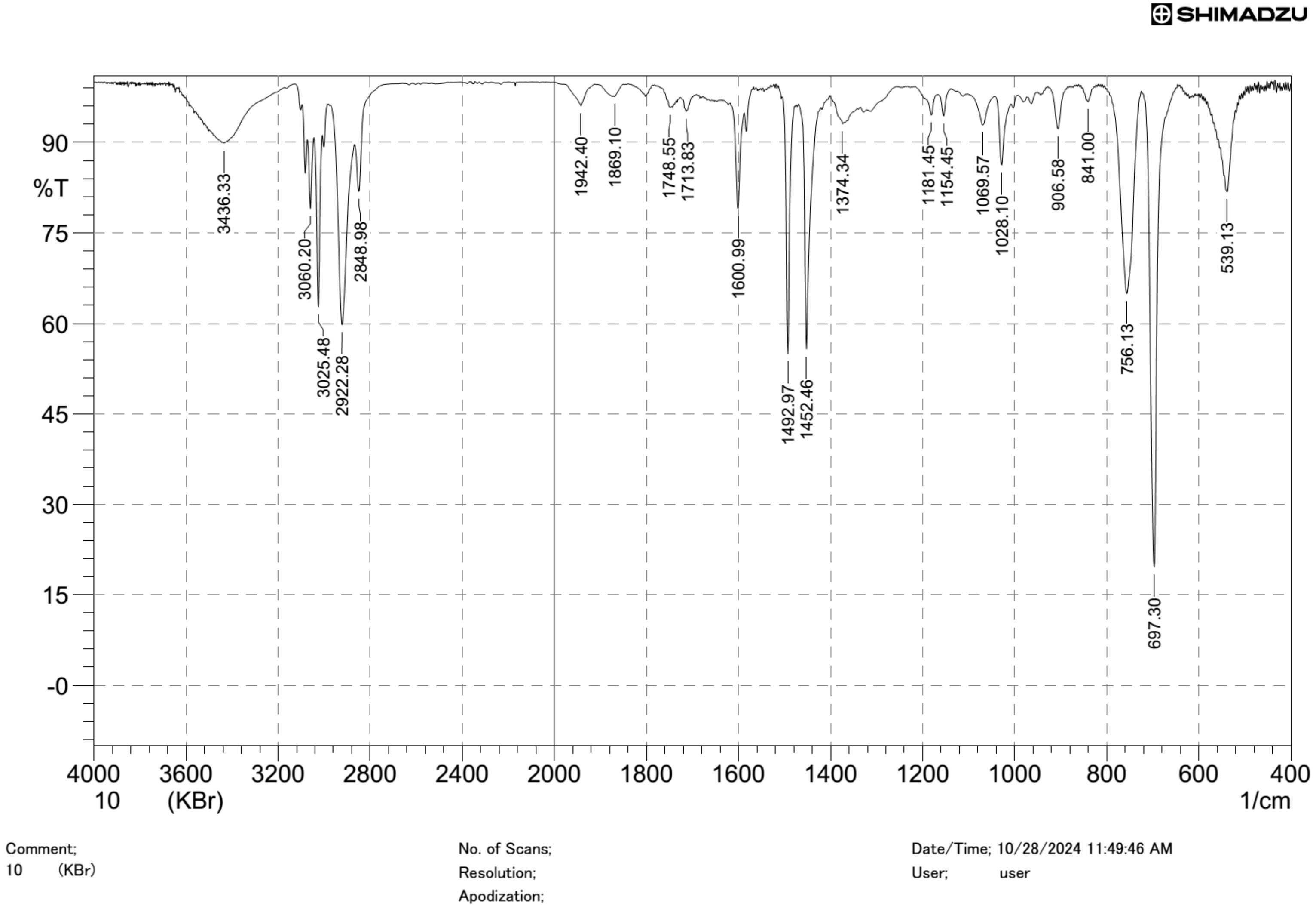

Figure S8: Original FTIR-spectra of model polystyrene particles PS3/9M +rhod.B
